# Zinc–Bromine Batteries: Challenges, Prospective Solutions, and Future

**DOI:** 10.1002/advs.202305561

**Published:** 2023-11-21

**Authors:** Asif Mahmood, Zhi Zheng, Yuan Chen

**Affiliations:** ^1^ School of Chemical and Biomolecular Engineering The University of Sydney Darlington NSW 2006 Australia; ^2^ Center for Clean Energy Technology School of Mathematical and Physical Science Faculty of Science University of Technology Sydney Sydney 2007 Australia

**Keywords:** aqueous batteries, Br_2_ cathodes, dendrite growth, flow/flowless batteries, zinc bromine batteries

## Abstract

Zinc‐bromine batteries (ZBBs) have recently gained significant attention as inexpensive and safer alternatives to potentially flammable lithium‐ion batteries. Zn metal is relatively stable in aqueous electrolytes, making ZBBs safer and easier to handle. However, Zn metal anodes are still affected by several issues, including dendrite growth, Zn dissolution, and the crossover of Br species from cathodes to corrode anodes, resulting in self‐discharge and fast performance fading. Similarly, Br_2_ undergoes sluggish redox reactions on cathodes, which brings several issues such as poor reaction kinetics, the highly corrosive nature of Br species leading to corrosion of separators and poisoning of anodes, and the volatile nature of Br species causing increased internal pressures, etc. These issues are compounded in flowless ZBB configuration as no fresh electrolyte is available to provide extra/fresh reaction species. In this review, the factors controlling the performance of ZBBs in flow and flowless configurations are thoroughly reviewed, along with the status of ZBBs in the commercial sector. The review also summarizes various novel methodologies to mitigate these challenges and presents research areas for future studies. In summary, this review will offer a perspective on the historical evolution, recent advancements, and prospects of ZBBs.

## Graphical abstract

Zinc‐bromine batteries (ZBBs) offer high energy density, low‐cost, and improved safety. They can be configured in flow and flowless setups. However, their performance and service still require significant improvement, particularly in flowless configurations. Recent advancements in electrode designs and electrolyte chemistries address some of these issues and pave the way for broader adoption in practical applications.

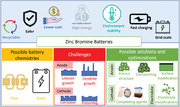

## 1 Introduction

Electrochemical energy storage devices are increasingly crucial in electrifying our society using renewable energy sources to replace fossil fuel‐based energy sources. Li‐ion batteries (LIBs) dominate applications from portable electronics, electric vehicles, and grid‐scale electricity storage.^[^ ^1^, ^2^, ^3^, ^4^ ^]^ The future development of LIBs faces challenges in a limited supply of required critical elements, such as Li and Co, and safety issues.^[^ ^5^ ^]^ Various aqueous batteries, such as Fe–Cr, Pb acid, Zn ion, Zn–Ce, and Zn–Br batteries (ZBBs), are attracting interest as alternatives to LIBs because they use earth‐abundant elements and offer better safety.^[^ ^6^, ^7^, ^8^, ^9^, ^10^, ^11^ ^]^ ZBB was proposed in 1885 and first commercialized in the 1970s by Exxon.^[^ ^12^ ^]^ ZBBs use low‐cost electrode materials (Zn and carbon) and have a high theoretical energy density of 428 Wh kg^−1^, comparable to LIBs.^[^ ^13^, ^14^, ^15^, ^16^, ^17^ ^]^ They may also offer high energy efficiency (≈65–90%) and voltage efficiency (≈83%).^[^ ^18^ ^]^ The advantages of high energy density, abundant elements, and safer operation have made ZBBs an attractive candidate for grid‐scale energy storage.

ZBBs usually use a metallic Zn anode, a carbon material cathode containing Br_2_ complexing agents, and an aqueous electrolyte containing ZnBr.^[^ ^19^, ^20^, ^21^ ^]^ Like other Zn‐based aqueous batteries, metallic Zn anodes in ZBBs suffer from dendrite growth, corrosion, and side reactions, resulting in gradual performance loss or sudden short‐circuiting.^[^ ^14^, ^15^, ^22^, ^23^, ^24^ ^]^ The redox reactions of Br_2_ at the cathode generate corrosive Br species, also causing performance degradation over time. Various carbon materials have been used in ZBBs. For example, carbon nanotubes (CNTs), graphene, and mesoporous carbon have been used in anodes to provide graphitic surfaces and homogeneous charge distribution, which enable reversible Zn stripping/plating and mitigate Zn dendrite formation.^[^ ^25^, ^26^, ^27^, ^28^, ^29^ ^]^ Carbon materials mixed with Br complexing agents in cathodes provide corrosion resistance, high electrical conductivity, and potentially a wider working voltage window.^[^ ^30^, ^31^ ^]^ The change in the working voltage window results from the beneficial effects of carbon materials in increasing the cathode surface area, facilitating rapid mass transport, and offering many active sites for redox reactions of Br species. For example, Wang et al. observed an increase in the working voltage window from 1.8 to 1.9 V when carbon materials were used.^[^ ^31^ ^]^ Electrolytes also strongly influence ZBBs’ performance.^[^ ^32^, ^33^, ^34^ ^]^ Theoretically, the voltage window of ZBBs should be the potential difference between Zn/Zn^2+^ and Br^−^/Br_2_. However, due to unwanted side reactions and corrosive Br species, the safe operating voltage range, often called the voltage window, could be smaller, which depends on the concentration of Br species in electrolytes. Further, the energy density of ZBBs also depends on various properties of electrolytes, such as the solubility of Br species, viscosity, and chemical stability. Various additives have been used to modify the properties of electrolytes to stabilize the ZBB performance. ZBBs have been primarily studied in flow battery configurations with liquid electrolyte reservoirs and pumps, making their operation complex. Their energy density is only ≈70 Wh kg^−1^, less than 20% of the theoretical energy density. Solidified electrolytes have also been explored. Flowless ZBBs have recently been demonstrated, opening a new research dimension.^[^ ^35^ ^]^

**Figure 1:**
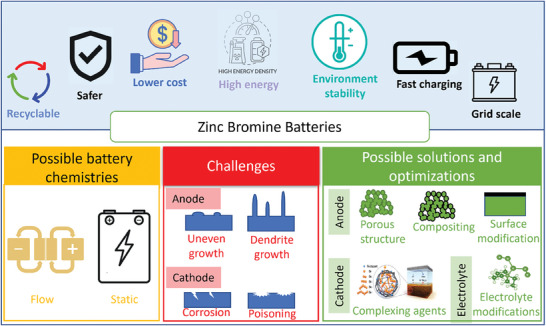
Schematic illustration of Zn‐Br battery's key advantages, possible chemistries, challenges, and room for further improvement.

In this review, we first introduce different configurations of ZBBs and discuss their status in scientific research and commercial development. Specifically, recent innovations reported in patents related to ZBBs are summarized. Next, we discuss technical challenges in improving their anode, cathode, electrolyte, and electrode/electrolyte interfaces. Then, we thoroughly summarize recent studies to address these challenges. The overall content of this review includes the critical advantages of ZBBs, their possible chemistries, significant challenges, and possible solutions, as illustrated in **Figure** **1**.

## 2 Current Status

Various Zn‐based aqueous batteries have been demonstrated, such as Zn–Fe, Zn–Ce, Zn‐I_2_, Zn‐air, and Zn–Br_2_,^[^ ^36^, ^37^, ^38^, ^39^, ^40^, ^41^ ^]^ indicating the versatility of Zn battery chemistry. Since all of them utilize Zn metal as their anode materials, their cost variance is primarily determined by their cathodes, electrolytes, and device configurations. For example, Zn flow batteries using V‐based cathodes/electrolytes can offer a high energy density of 15–43 Wh L^−1^; however, the high cost of V (US$ 24 per kg) limits their commercial‐scale adoption.^[^ ^12^, ^42^ ^]^ Zn flow batteries using Fe‐based cathodes/electrolytes (US$ 0.8 per kg) are a low‐cost alternative; however, Zn–Fe batteries have a low energy density.^[^ ^12^ ^]^ ZBBs are attractive because Br‐based cathodes/electrolytes are relatively cheap (US$ 2 per kg). At the same time, they offer a high energy density of up to 65 Wh kg^−1^, as listed in **Table** **1**.^[^ ^12^ ^]^ Moreover, ZBBs provide a high cell voltage of 1.8 V.

**Table 1:** Costs and energy densities of different types of aqueous flow Zn batteries. (Adapted from Khor et al.^[^ ^12^ ^]^)

Flow batteries	Cell voltage (open‐circuit)/V	Negative electrode cost (element)/USD$ kg^−1^	Positive electrode cost (element)/US$ per kg	Electrolyte cost/US$ per [kW h]*	Typical energy density/W h L^−1^
Zn‐Fe	1.5	1.9	0.8	5	‐
Zn‐Ce	2.4	1.9	12	42	12–20
Zn‐I_2_	1.3	1.9	13.5	41	Up to 1.1
Zn‐organic (Benzoquinone)	1.6	1.9	5	14	2–5
Zn‐air	1.6	1.9	Nil	4	‐
Zn‐Br_2_	1.8	1.9	2.0	5	Up to 65
Zn‐V	1.4	1.9	24	87	15–43
All‐Organic (Viologen‐TEMPO)	1.2	5	5	92	0.5–5

Considering the long history of ZBB chemistry, Section 2.1 summarizes their current status: battery chemistry and two types of battery configurations. The commercial development and an overview of essential patents are discussed in Section 2.2.

### 2.1 Battery Chemistry

ZBBs have four main components: two electrodes, electrolytes, and a membrane separator. The main electrochemical reactions on their cathodes and anodes are listed below (standard hydrogen electrode (SHE):

(1) $${\mathrm{Zn}}^{2+}+2e^{-}↔\mathrm{Zn}\;\;E_{o}=-0.76\;V\;\mathrm{vs}\;\mathrm{SHE}\left(\mathrm{Negative}\right)$$

(2) $$2{\mathrm{Br}}^{-}↔{\mathrm{Br}}_{2}+2e^{-}\;\;E_{o}=+1.07\;V\;\mathrm{vs}\;\mathrm{SHE}\;\left(\mathrm{Positive}\right)$$

(3) $$2\mathrm{ZnBr}↔\mathrm{Zn}+{\mathrm{Br}}_{2}\;\;E_{o}=+1.83\;V\;\mathrm{vs}\;\mathrm{SHE}\;\left(\mathrm{Overall}\right)$$

**Figure 2:**
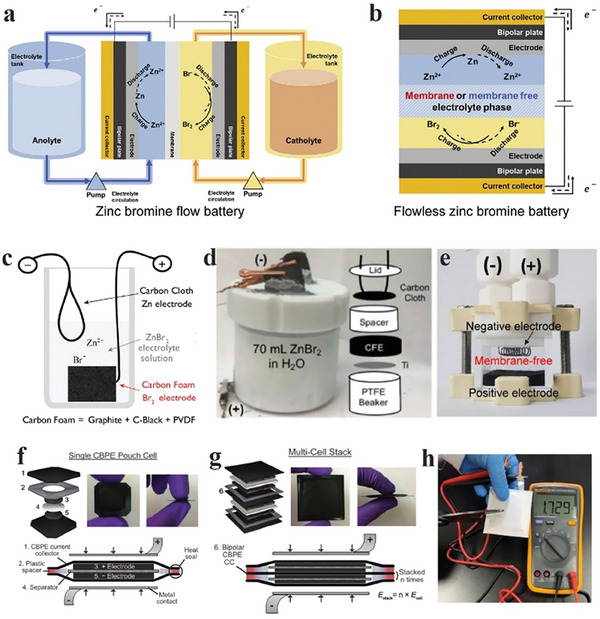
Different structures of a) conventional ZBFB and b) FL‐ZBB, reproduced with permission.^[^ ^14^ ^]^ Copyright 2022, Elsevier. c) Schematic illustration of an FL‐ZBB hosted in a beaker, reproduced with permission.^[^ ^55^ ^]^ Copyright 2017, the Royal Society of Chemistry. d) A photo and schematic of an FL‐ZBB cell hosted in a PTFE bottle, reproduced with permission.^[^ ^57^ ^]^ Copyright 2018, the Institute of Physics. e) A photo of an FL‐ZBB cell without a separation membrane before charging, reproduced with permission.^[^ ^58^ ^]^ Copyright 2019, Wiley‐VCH. f,g) Single and stacked FL‐ZBB pouch cells: f) Cross sectional views and photos of a single pouch cell comprised of freestanding electrodes and a separator between two current collectors surrounded by an insulating plastic spacer. g) Cross sectional views and photos of a bipolar pouch cell with two cells assembled in series, reproduced with permission.^[^ ^60^ ^]^ Copyright 2018, the Royal Society of Chemistry. h) A photo of an FL‐ZBB pouch cell, reproduced with permission.^[^ ^35^ ^]^ Copyright 2020, Cell Press.

During charging, Zn^2+^ in electrolytes is reduced to metallic Zn on the surface of an anode. Br^−^ in the electrolyte is oxidized to Br_2_ molecules on a cathode, where Br_2_ molecules and Br^−^ are further complexed into polybromides by a sequestration agent.^[^ ^30^, ^43^ ^]^ During discharging, the reverse reactions occur, and metallic Zn is oxidized into Zn^2+^, entering the electrolyte. Br_2_ molecules are reduced to Br^−^. The electrochemical Br_2_ redox reaction (Br_2_/Br^−^) is much more sluggish than the Zn redox reaction (Zn^2+^/Zn). The standard rate constant for the Br_2_ redox reaction (Br_2_/Br^−^) is 4 × 10^−7^ m s^−1^, while the rate constant for the Zn redox reaction (Zn^2+^/Zn) is 7.5 × 10^−5^ m s^−1^.^[^ ^44^ ^]^ Based on the same battery chemistry, ZBBs have been developed in flow and flowless configurations, as illustrated in **Figure** **2**a,b. In the following two subsections (2.1.1 and 2.1.2), ZBBs in these two configurations are briefly described.

#### 2.1.1 Flow ZBBs

In addition to two electrodes, electrolytes, and a separator, Zn‐Br flow batteries (ZBFBs) require two sets of electrolyte reservoirs and pumps to ensure the efficient flow of Zn and Br‐containing electrolytes during battery operation.^[^ ^38^, ^45^, ^46^ ^]^ The performance of ZBFBs depends on the stability of their Zn anode and Br_2_‐containing cathode. Two external reservoirs provide fresh electrolytes to increase or enhance cell life significantly. Historically, metallic Zn anodes have been used in ZBFBs; however, they are prone to quick degradation because of the fast growth of dendrites and the dissolution of Zn in electrolytes. Porous carbon has been used as a host for Zn anodes; however, such carbon/Zn composite anodes often can only enable low current densities.^[^ ^47^, ^48^ ^]^ Many electrode modification strategies have been proposed to address these Zn anode challenges, which are summarized in later sections.

Br_2_ in cathodes undergoes reversible complexation reactions during charging/discharging,^[^ ^30^, ^49^ ^]^ which requires a high polarization, leading to low current power densities. Polarization in batteries is a voltage difference between expected and actual performance due to internal resistance and ion transport limitations, reducing efficiency and capacity while generating heat. Therefore, a high polarization in ZBBs due to the Br_2_ cathode can lead to lower energy density and efficiency. Moreover, Br^−^ in the cathode side may crossover to the anode side, poisoning the metallic Zn electrode. Many studies have reported different approaches to balance the reaction rates of the Br_2_/Br^−^ and Zn^2+^/Zn redox reactions to improve ZBFBs’ reversibility and power density.^[^ ^29^, ^31^, ^50^, ^51^, ^52^ ^]^ For example, incorporating porous carbon materials in cathodes has significantly increased Br_2_ cathodes' performance.^[^ ^29^, ^53^ ^]^ Similarly, various electrolyte and separator modifications have also been reported to improve the performance of ZBFBs, which are summarized in later sections.

#### 2.1.2 Flowless ZBBs

The first flowless ZBB (FL‐ZBB) was reported in 1995, in which the removal of pipelines, electrolyte reservoirs, and pumps was suggested.^[^ ^54^ ^]^ However, it was not until recently that rechargeable FL‐ZBBs attracted a broader interest. Biswas et al. reported an FL‐ZBB with a simple biker architecture without the electrolyte reservoirs and pumps, as shown in Figure 2c.^[^ ^55^ ^]^ The authors compared the cost reduction using the levelized cost of energy stored ($/kWh/cycle/%) and suggested ≈$0.017 for FL‐ZBBs, much lower than ≈$0.052 for ZBFBs and ≈$0.58 for LIBs. Their findings suggested the commercial viability of FL‐ZBBs. Knehr et al. also presented a series of studies demonstrating FL‐ZBBs in a beaker‐type configuration (Figure 2d).^[^ ^56^, ^57^ ^]^ The authors used a polytetrafluoroethylene (PTFE) bottle instead of a glass bottle, decreasing ohmic resistance and enhancing energy efficiency. They also found that Ti current collectors and metallic Zn anodes would lead to poor stability.

Several other FL‐ZBB configurations have also been proposed lately. Lee et al. presented a membrane‐less ZBB in an enclosed container, as shown in Figure 2e.^[^ ^58^ ^]^ The battery was charged at a current density of 5 mA cm^−2^ and delivered an energy density of 7.8 Wh L^−1^ with excellent stability for over ≈ 1000 cycles and energy efficiency of over 80%. They estimated a cost of $ 0.013/kWh/cycle, 23% lower than that presented by Biswas et al.^[^ ^59^ ^]^ Going one step further, Evanko et al. presented FL‐ZBB pouch cells with excellent electrochemical performance, as shown in Figure 2f,g.^[^ ^60^ ^]^ Tetraethylammonium bromide was utilized along with activated carbon to mitigate the challenges with the cathode and achieved a high cell‐level energy density of 50 Wh/L at a scan rate of 10 C. The FL‐ZBBs lost only ≈1% capacity over a long cycling of 500 cycles. They stacked four cells together to operate in the 6–7 V voltage window. In another report by Gao et al., an FL‐ZBB pouch cell was demonstrated using an additive (tetrapropylammonium bromide (TPABr)), which mitigated the cross‐diffusion of Br species and stabilized the anode interface, as shown in Figure 2h.^[^ ^35^ ^]^

Even though increased attention has been devoted to FL‐ZBBs in the last few years, the commercial development of FL‐ZBBs is still at an early stage. It is still unclear how FL‐ZBBs would behave at the whole‐cell level, what specific energy densities could be achieved, and their cycling stability. The following section (2.2) discusses ZBBs’ current commercialization status.

### 2.2 Current Commercialization Efforts

Zn‐based batteries have been commercialized with various chemistries, such as Zn–carbon, Zn‐air, Zn–Li, Zn–Ni, Zn–Ag, and Zn‐MnO_2_ batteries.^[^ ^61^ ^]^ Most of these batteries are either primary (not rechargeable) or flow batteries, currently produced in large quantities by Panasonic, Zincell, Xiamen 3 Circles Battery, Primus Power, and EOS Energy Storage. Companies, such as Salient, Zinium, Tuscan Tech, EOS Energy Storage, Aza, AEsir, and Gelion, have commercialized Zn‐based secondary batteries. The development of ZBFBs was started by Lim et al. in 1977.^[^ ^62^ ^]^ ZBFBs have been demonstrated in numerical large‐scale commercial applications. For example, VionX Energy demonstrated ZBFB installations with a storage capacity of 0.5 MW.^[^ ^12^ ^]^ Similarly, a 500 kWh ZBFB was installed at the Illinois Institute of Technology, USA. In 2019, Gelion Technologies, led by Maschmeyer et al., commercialized the first FL‐ZBB. Representative companies that have commercialized ZBBs are summarized in **Table** **2**.

**Table 2:** Existing installations and applications of ZBBs (Data adapted from Ref. [12]) and key patents related to ZBBs.

Company/Organization	Customer	Specification	Application	Installation year
Ensync Energy Systems (Previously ZBB Energy)	Detroit Edison, USA	400 kWh	Load levelling	2001
	United Energy, Melbourne, Australia	200 kWh	Demonstration of network storage applications	2001
	Nunawading Electrical Distribution Substation, Box Hill, Australia	400 kWh	Load levelling	2001
	Australian Inland Energy, Australia	500 kWh	Solar energy	2002
	Power Light, USA	2 × 50 kWh	Solar energy	2003
	Pacific Gas and Electric Co., USA	2 MWh	Peak power capacity	2005
	Dundalk Institute of Technology, Ireland	125–500 kWh	Wind energy	2008
	Illinois Institute of Technology, USA	500 kWh	Microgrid	2014
	Fort Sill, Oklahoma, USA	500 kWh	Microgrid	2013
Kyushu Electric Power & Meidensha	Kyushu Electric Power, Japan	1 MWh/4 MWh	Electric‐utility applications	1990
Redflow	University of Queensland, Australia	12 × 120 kW h	Solar energy storage	2011
Department of Energy, USA	Albuquerque, New Mexico, USA	2.8 MW h	Solar energy storage	2011
VionX Energy (Premium Power)	Massachusetts, USA	0.5 MW/3 MW h	Peak power capacity	2016
Gelion Technologies	Sydney, Australia			
Essential patents related to ZBBs				
Year	Patent No	Inventor	Key Innovations	Ref
1951	2640864USA	Adolph Fischbach et al.	Electrode materials: a two‐step calcination method to produce porous Zn. Short exposure of Zn pastes at 300°C followed by calcination between 500–700 °C for 10–15 s	[79]
1974	3806368USA	Donald L. Maricle et al.	Electrolytes: salts as an additive to stabilize Zn‐based batteries.	[63]
1984	83306866.1	Ralph Zito	Cell design: several individual cells are connected in series to promote cell stability and 0.5–1.5 m electrolyte for charging while 2–4 m electrolyte for discharging	[64]
2006	20060273005A1	Charles Love et al.	Electrode materials: two types of inorganic particles merged in a slurry to introduce porosity	[80]
2007	007291186B2	Xiaoge Gregory Zhang	Electrode materials: forming flexible and densified Zn structures by molding and pressing Zn filaments, fibres, threads, or strands together at room temperature	[70]
2016	WO2016/057457A2	George W. Adamson et al.	Bipolar electrodes: cathodes with holes for improved electrolyte diffusion	[66]
2017	20170338479A1	Joseph F. Parker et al.	Electrode materials: forming porous Zn foams by fusing and merging Zn particles slightly below the melting point of Zn followed by calcining between 600–700°C and electrochemical reduction	[81]
2017	WO2017/070340A1	Wei Xia et al.	Electrolytes: adding ammonium halide‐based salts as an additive	[69]
2017	US2017/0025677A1	Daniel A. Steingart et al.	Electrode materials: forming porous Zn structures with porosity close to 80% by electrodeposition in a highly concentrated KOH (9 M) solution and ≈0.6 M ZnO	[73]
2018	WO2018/071469A1	Daniel A. Steingart et al.	Cell design: membrane‐free ZBBs with carbon‐based anodes and cathodes suspended in electrolytes.	[68]
2019	US2019/0097273A1	Thomas Maschmeyer et al.	Electrolytes: gelated electrolytes	[78]
2020	WO2020086838A1	Jinchao Huang et al.	Electrode materials: Zn expanded mesh with the precise control of areal porosity between 0.7–3.5 g cm^−2^.	[82]
2020	US10720635B2	Debra R. Rolison et al.	Electrode materials: forming porous Zn by stepwise calcination and using solid and liquid phases to achieve a highly porous structure	[83]
2021	US20210020916A1	Brandon J. Hopkins et al.	Electrode materials: using double porogens to control the porosity of Zn foams, one porogen would sublimate at a lower temperature, and the other decomposes at a higher calcination temperature	[72]
2022	US2022/0059846A1	Thomas Maschmeyer et al.	Electrolyte: modifying electrolyte composition using halogen sequestering agents	[77]

The development of commercial ZBBs has been described in several patents and briefly reviewed here (Table 2). The early patents published in the 1970s and 1980s focused on the design of ZBFBs.^[^ ^63^, ^64^ ^]^ These ZBFBs had an anode and a cathode connected with electrolyte reservoirs.^[^ ^65^, ^66^, ^67^ ^]^ Maricle et al. invented a secondary ZBB in 1974 using the ZnBr electrolyte and a non‐reactive additive to stabilize the assembled ZBBs.^[^ ^63^ ^]^ Similarly, Zito et al. reported a stacked ZBB design in 1984 using a single recirculating electrode coupled with an external storage tank.^[^ ^64^ ^]^ These inventions paved the way for ZBFB commercialization. Some patents proposed different battery configurations. For example, Steingart et al. from Princeton University invented a simple membrane‐free method that utilized a carbon cloth as a host for Zn anode suspended in 2 m ZnBr_2_ electrolyte and a carbon foam as a Br_2_ electrode settled at the bottom of the electrolyte container.^[^ ^68^ ^]^

Apart from the patents describing overall battery cell designs, a range of patents focused on strategies to stabilize Zn metal anodes, Br_2_ cathodes, and electrolytes, either individually or in combinations. Several patents described Zn electrode fabrication methods to improve their cycling life and charge storage capacity.^[^ ^69^, ^70^, ^71^, ^72^, ^73^ ^]^ For example, Zhang et al. invented a way to produce porous Zn electrodes by compressing Zn fibers, threads, and filaments.^[^ ^70^ ^]^ Hopkin et al. from the USA Navy Research Lab filed a series of patents on forming 3D Zn foams using Zn powders.^[^ ^71^ ^]^ Their methods utilized precursors with different chemical compositions to control the porosity of Zn foams. High‐temperature calcination was used to fuse Zn particles into monolithic structures. In their first patent, Hopkins et al. described a 3‐step method. Zn particles mixed with polymeric surfactants were first merged into a monolithic form by heating in an Ar environment. Next, the fused Zn structure was calcined in the air to remove surfactants, which otherwise would introduce an unwanted layer of ZnO. Last, an electrochemical reduction step was applied to reduce surface ZnO back to metallic Zn. The follow‐up patent from this team reported several modifications to the original 3‐step method. For example, a different set of polymeric additives was used, which were removed by sublimation to generate the porosity in the Zn foam. The reduction step for the surface ZnO layer was also removed because it can be achieved during battery charging/discharging.

Several other patents focused on stabilizing Br_2_ cathodes via electrode structural designs or chemical composition.^[^ ^66^ ^]^ For example, Adamson et al. from EOS Energy Storage filed a patent on using a Ti cage to hold carbon materials in cathodes.^[^ ^66^ ^]^ The Ti cage ensured the cathodes’ mechanical strength and reduced contract resistance. This cathode design was applied to different batteries that used halides as cathode materials. Another patent from Adamson et al. described cup‐shaped metal current collectors used in bipolar battery stacks, which provided uniform current flow through batteries.^[^ ^74^ ^]^ Some patents have also been filed on different electrolyte compositions. Magnes et al. from Bromine Compounds filed a patent on complexing agents for Br_2_ in ZBBs.^[^ ^75^ ^]^ Complexing agents, such as l‐n‐butyl‐2‐methyl‐pyridinium, l‐butyl‐2‐methyl pyridinium bromide, would form a water‐immiscible phase and settle in the electrolyte reservoir. This process can act reversibly upon demands. Ge et al. from Albemarle Corporation filed a patent related to the use of quaternary ammonium halide (*A* [(*CH*_2_)_*E*_ − *O* − (*CH*_2_)_*F*_]_*n*_ *B*).^[^ ^76^ ^]^ It was claimed that the chemistry and stability of the complexing agent could be readily adjusted by changing the composition of A and B while keeping their primary chain identical.

Very recently, the focus of new patents shifted toward FL‐ZBBs. Maschmeyer et al. from Gelion Technologies filed patents on halogen sequestering agents.^[^ ^77^ ^]^ The structure of the sequestering agent is as shown in Equation (4), where Z could be any of N, P, or S, while R^1^, R^2^, R^3^, and R^4^ are organic groups depending on different requirements. It was claimed that quaternary ammonium, phosphonium, or sulfonium halides could be used in ZBFBs and FL‐ZBBs. Flexibility in the structure enables tunability. Another patent filed by Gelion Technologies relates to a gel electrolyte made of ionic liquids. The gel electrolyte was claimed to replace liquid electrolytes for FL‐ZBBs.^[^ ^78^ ^]^


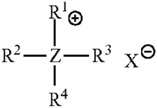
(4)

## 3 Key Technical Challenges

ZBBs are considered hybrid batteries based on their energy storage mechanism.^[^ ^84^ ^]^ This section will summarize critical technical challenges in their key components, including anodes, cathodes, electrolytes, and electrode/electrolyte interfaces.

### 3.1 Challenges of Zn Anodes

**Figure 3:**
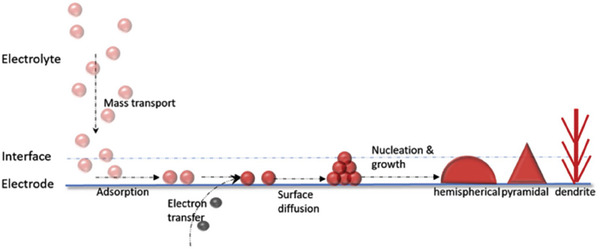
A schematic illustration shows the major steps of metal electrocrystallization. Reproduced with permission.^[^ ^16^ ^]^ Copyright 2020, Elsevier.

Zn anodes play a critical role in determining the cycling life of ZBBs because they are prone to uneven Zn deposition during cycling, leading to dendrite growth and Zn dissolution into the electrolyte. The stripping/plating of Zn is governed by the Zn/electrolyte interface, which determines the homogeneity of Zn deposition. Zn plating has four distinct phases, i.e., Zn^2+^ diffusion, reduction, nucleation at the interface, and subsequent crystal growth, as shown in **Figure** **3**.^[^ ^16^, ^23^, ^61^, ^85^ ^]^ Theoretically, homogenous Zn plating is achievable if local conditions of the Zn/electrolyte interface are uniform. However, the local conditions, such as the electrical charge distribution and surface roughness, are non‐uniform, forming dendrites with hemispherical, pyramidal, or needle structures. Some dendrites could pierce the separator and short‐circuit the ZBB. Dendrite formation can be slowed to some extent by limiting the Zn ion concentration gradient, enhancing Zn electrode electrical conductivity, tuning electrolyte composition, and raising the operating temperature.^[^ ^86^ ^]^

The growth of dendrites can be divided into three primary steps i) growth, ii) dissolution, and iii) regrowth.^[^ ^16^ ^]^ Freshly prepared electrodes may offer homogeneous charge distribution initially. However, Zn metal is not entirely stripped off during the stripping upon repeated cycling. Some metal residues left behind create preferential sites for further nucleation and subsequent cycling, eventually leading to Zn growth in certain areas as islands. The islands further grow into needle‐like dendrites. In addition, an electrode's surface chemistry and roughness also play a critical role where an electrode surface with higher roughness causes inhomogeneous current density distribution.

**Figure 4:**
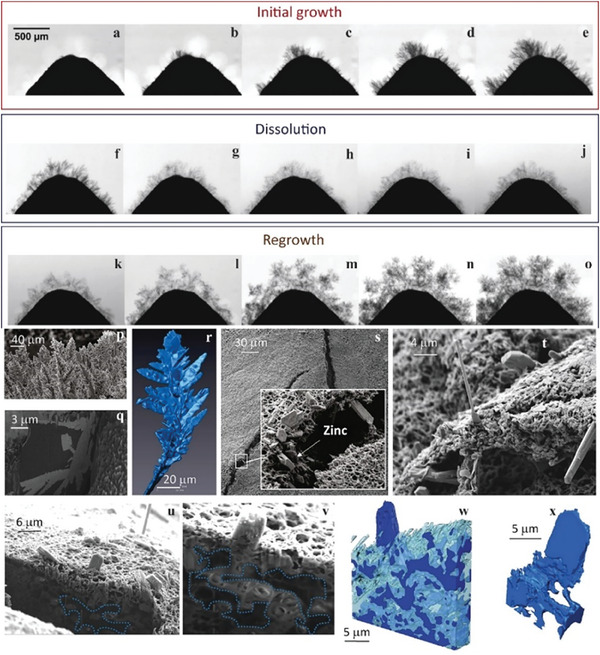
An operando study of Zn dendrite growth, dissolution, and regrowth in a cell without a separator under a current density of 30 mA cm^2^. a–o) Zn dendrite growth after a) 214 s, b) 388 s, c) 561 s, d) 734 s, and e) 918 s. Zn dendrite dissolution after f) 10 s, g) 229 s, h) 447 s, i) 655 s, and j) 873 s. Zn dendrite regrowth after k) 122 s, l) 589 s, m) 1028 s, n) 1485 s, and o) 1953 s. SEM images of Zn dendrites grown at 30 mA cm^−2^ p,q): p) deposited dendrites, and q) cross sectional view of a single dendrite embedded in epoxy. r) a 3D‐reconstructed image of a single dendrite, s) SEM images of a separator torn by dendrites, t) a dendrite structure grown through the separator, u) a cross sectional view of dendrites grown inside the separator, and v) a cross sectional view of Zn deposits in the separator outlined by dotted lines connected to the dendrites on the top. w) A 3D‐reconstructed image of the Zn dendrite attached to Zn deposits (dark blue) grown inside the porous separator (transparent light blue), and x) a 3D image of the single dendrite grown from the separator. Reproduced with permission.^[^ ^87^ ^]^ Copyright 2019, Cell‐press.

Yufit et al. performed an operando study to monitor dendrites' growth thoroughly. They found that electrode/electrolyte interfaces strongly influence the charge concentration on the electrode surface and the overall charge transfer.^[^ ^87^ ^]^ The growth of a needle‐like structure was observed during the initial plating, as shown in **Figure** **4**a. With the extension of plating time, the needle‐like structure grew into a porous tree‐like structure, as shown in Figure 4b–e. The dendrite growth was accompanied by the hydrogen evolution reaction (HER). Subsequently, the dendrite dissolved during stripping (Figure 4f–j). The stripping step did not strip off Zn completely from the electrode surface. The left‐behind residues provided nucleation sites for dendrite growth in the next charging cycle, resulting in a denser structure (Figure 4k–o). Apart from the electrode microstructure, the crystalline structure of Zn electrodes can also impact Zn plating behavior.^[^ ^88^ ^]^

The structure of dendrites was also carefully examined by ex‐situ characterization tools. Focused ion beam scanning electron microscope (FIB‐SEM) images showed a “forest” of spruce tree‐like shapes on the Zn electrodes when Zn was deposited under mass transport limitations (Figure 4p). The dendrites are dense, while their surfaces show a variation in contrast (Figure 4q). The contrast variation indicates different crystalline structures. A simulated dendrite was regenerated, as shown in Figure 4r. SEM images also show that needle‐like structures (≈800 nm thick) pierce through the separator, creating large cracks up to ≈15 um (Figure 4s,t). It was also found that the dendrites embedded inside the separator have a different morphology than those on the electrode surface (Figure 4u,v). The dendrites grew into the pores of the separator through open spaces rather than growing in a straight needle‐like structure on the electrode surface. Figure 4w,x shows 3D‐reconstructed dendrites in a separator.

HER on the Zn anode surface presents another significant issue. This involves the unintended production of H_2_ gas during the battery's operation, which can be attributed to factors such as overpotential, impurities within the electrolyte, or catalytic reactions. H_2_ gas generation poses dual concerns regarding safety due to its flammability and efficiency due to its energy consumption, ultimately diminishing the battery's overall performance. Strategies for mitigating this problem encompass enhancing cathode design, ensuring electrolyte purity, and incorporating mechanisms for pressure relief. Effectively addressing this concern is pivotal for the broader adoption of ZBBs in grid‐scale energy storage and the integration of renewable energy sources, given their potential for scalability and extended cycle life.

Zn metal foils have been commonly used as anodes in ZBBs. However, Zn metal foils are prone to rapid corrosion in acidic electrolytes containing corrosive Br_2_. More stable metals, such as Ti, have been explored to replace Zn. However, they can also be oxidized or brominated.^[^ ^89^ ^]^ Recent efforts include carbon‐based composites and highly porous Zn foams, which are summarized in subsequent sections.

### 3.2 Challenges of Cathodes

**Figure 5:**
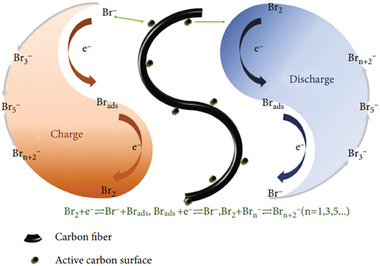
The mechanism of Br_2_/Br^−^ reactions on the cathode in ZBB. Reproduced with permission.^[^ ^30^ ^]^ Copyright 2022, American Association for Advancement of Science.

ZBB cathodes use a substrate to catalyze the Br_2_ redox (Br_2_/Br^−^) reaction.^[^ ^30^ ^]^ Br_2_ has a solubility of 0.43 mol L^−1^ in water. With significantly increased solubility, Br_2_ makes a strong complex with halogen ions to form Br_3_^−^/Br_5_^−^. The high solubility of Br species may lead to a crossover to Zn anodes, where Br_2_ reacts with Zn, lowering Coulombic efficiency (CE).^[^ ^30^, ^31^, ^90^, ^91^ ^]^ The mechanism of Br_2_ redox couple reaction is summarized in **Figure** **5**.

Cathodes in ZBBs are suffering from several issues. First, Br_2_ is highly corrosive and may quickly degrade separators. Separators are porous membranes that keep battery electrodes apart while allowing ion flow. Due to its highly corrosive nature, Br_2_ can harm these separators, shortening battery lifespan, reducing performance, and posing safety risks. Br_2_/Br^−^ species can crossover to anodes, resulting in battery self‐discharge. To tackle this issue, design strategies are presented in the electrolyte and prospect sections. Second, the Br_2_ redox (Br_2_/Br^−^) reaction is sluggish, resulting in high polarization and low power density. Third, Br_2_ has high volatility, leading to increased gas pressure in ZBBs, causing safety issues. Understanding the Br_2_ redox reaction and polybromide nucleation is essential to achieving stable cathodes. Han et al. used confocal microscopy to study Br_2_ complexation during its redox reaction. They showed Br_2_ emulsion droplet formation when the oxidative potential was applied.^[^ ^92^ ^]^ The droplets grew larger with the increase of applied potential and subsequently detached from electrode surfaces. Some detached droplets moved back toward the electrode surface, causing a spike in the current. However, the limited resolution of confocal microscopy makes it challenging to differentiate between emulsion droplets and electrode surfaces.

**Figure 6:**
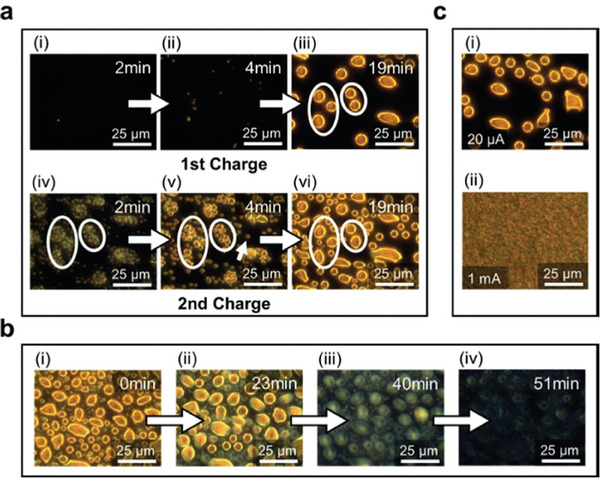
a) i–iii) Operando time‐lapse images obtained by dark‐filed light microscopy show liquid polybromide droplet formation in the first cycle. iv–vi) Images at the same location during charging in the second cycle. The white arrow in v) indicates the emerging of a droplet at a new location. The white circles in iv–vi) indicate the droplet growth from residuals at the same locations between cycles. b) Time‐lapse images of microdroplets disappearing upon resting (without applying potentials). c) Comparison of droplets formed with low (i) and high (ii) charging current density to the same capacity. The low current density produces sparse and large droplets, while the high current density produces dense and small droplets. Reproduced with permission.^[^ ^93^ ^]^ Copyright 2019, Wiley‐VCH.

Alternatively, Wu et al. utilized an operando visualization method to monitor polybromide formation.^[^ ^93^ ^]^ Liquid polybromide droplets strongly interacted with electrode surfaces. These droplets were not completely removed during repeated charge/discharge cycles. The residues on electrode surfaces provide nucleation sites to grow droplets further, as shown _by_ white circles in **Figure** **6**a,b. A spontaneous polybromide dissolution in the electrolyte was observed without applying potentials (Figure 6b). The time of the spontaneous dissolution process was found to be proportional to the charge capacity of the battery. The crossover of the polybromide ions to Zn anodes and subsequent reactions caused an imbalance in the stoichiometric concentration of polybromide in the electrolyte, leading to more polybromide dissolution. Further, the applied current density can regulate polybromide droplets' size and reaction rate. Large diameter (5–10 um) droplets were observed under a low current density (0.066 mA cm^−2^) (Figure 6c). Smaller diameter (2–3 um) droplets were observed under a higher current density (1 mA, 3.33 mA cm^−2^). It was proposed that the nucleation rate was directly proportional and the nucleation radius was inversely proportional to the current density.

### 3.3 Challenges of Electrolytes

**Figure 7:**
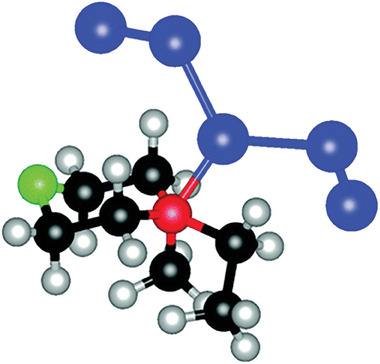
A proposed structure (optimized via periodic density functional calculations) of N‐methyl N‐ethyl morpholinium bromide and two sequestered Br_2_ molecules. Black: carbon, gray: hydrogen, red: nitrogen, blue: bromine, and green: oxygen.^[^ ^97^ ^]^

Electrolytes play a crucial role in determining the energy density and voltage window of ZBBs. Traditionally, ZBBs use an aqueous ZnBr_2_ solution as electrolytes with a molarity of 1–3 m. Higher concentrations of ≈4 m have also been used.^[^ ^94^ ^]^ Various additives, such as KCl, Zn(ClO_4_)_2_, and NH_4_Cl, have been used to improve the electrochemical performance of ZnBr_2_ electrolytes.^[^ ^62^, ^95^, ^96^ ^]^ Toxic and corrosive Br_2_ gas may be formed during charging/discharging, and it can crossover to anodes to corrode Zn metal electrodes. Thus, complexation agents have been used to minimize the formation of Br_2_ gas. Quaternary bromide salts, such as N‐methyl N‐ethyl pyrrolidinium bromide (MEPBr) and N‐methyl N‐ethyl morpholinium bromide (MEMBr), are commonly used complexation agents (**Figure** **7**). Recent studies have also explored quasi‐solid (gel) or solid electrolytes to mitigate the problems of liquid electrolytes. They have the potential to provide a better barrier in preventing Br_2_ crossover and offer a wider voltage window. However, solidified electrolytes have a lower ionic conductivity than liquid electrolytes, resulting in higher ohmic polarization. The poorer interfaces between solidified electrolytes and electrodes also affect the charge transfer.

### 3.4 Challenges of Electrode/Electrolyte Interfaces

**Figure 8:**
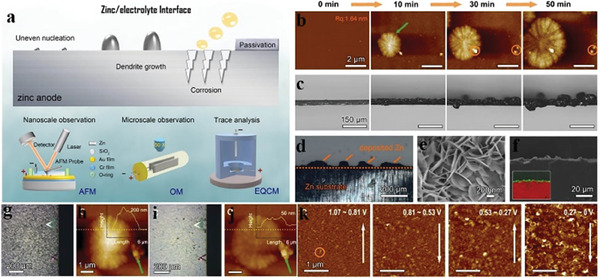
a) Schematic illustration of issues at Zn/electrolyte interfaces and a multiscale in situ characterization system to study the nucleation and dendrite growth of Zn in 1.0 m ZnSO_4_ electrolyte. b) AFM images and c) optical cross sectional images of Zn deposition at different deposition times. d) A plane‐view optical image of deposited Zn in (c). e) A top view SEM image and f) a cross sectional SEM image of deposited Zn on graphite; the inset shows the elemental mapping of Zn (green) and C (red). g) Optical and h) AFM images of deposited Zn and i) optical and j) AFM images under the quasi‐zero electrochemical field for 10 min; the insets in AFM images show the corresponding height information. k) In situ potential‐dependent AFM topography images. The long white arrows indicate the AFM scan directions. Reproduced with permission.^[^ ^98^ ^]^ Copyright 2020, American Chemical Society.

The challenges of electrode/electrolyte interfaces have been partially covered in the preceding sections. Here, their roles in determining the reversibility of the ZBBs are further highlighted. As described previously, the stability of Zn anodes is severely affected by inhomogeneous deposition, dendrite growth, corrosion, and passivation, as shown in **Figure** **8**a. These detrimental issues are all related to electrode/electrolyte interfaces. The surface chemistry of electrodes, electrolyte composition and pH, and applied voltages can impact the interfaces. Zhou et al. combined in situ atomic force microscopy (AFM), optical microscopy, and electrochemical quartz crystal microbalance (Figure 8a) to reveal various changes at the interface between aqueous electrolytes and Zn anodes.^[^ ^98^ ^]^ Zn electrodes with a surface roughness of ≈1.64 nm were used to study Zn deposition under a current density of 0.04 mA cm^−2^ for 50 min, as shown in Figure 8b. Zn deposition started at specific sites and then grew anisotropically. The Zn deposits grew as dense semi‐spherulites rather than filamentous or moss shapes of Li/Na deposits. Optical images show that Zn preferably deposits at electrode edges (Figure 8c,d). Their SEM images show a flower‐shaped morphology (Figure 8e) and Zn dendrite formation (Figure 8f). The self‐corrosion of Zn deposits was studied using optical microscopy and AFM. Optical images in Figure 8g,i show slow corrosion of Zn under a quasi‐zero electrochemical field. The corresponding AFM images show a large Zn particle before applying a quasi‐zero electrochemical field (Figure 8h), which fades away partially after some time (Figure 8i). Figure 8k further reveals the real‐time changes of the Zn surface upon applying an electrochemical potential, where tiny flake‐like structures grew in height to ≈12 nm when the potential approached 0 V.

## 4 Methodologies to Achieve High Performance

Recent years have seen tremendous progress in improving ZBBs’ performance. Research efforts have been devoted to improving all components in ZBBs, including i) improving anodes to enable homogeneous Zn stripping/plating, ii) modifying cathodes to improve their catalytic activity for Br redox reactions and trapping capability of Br species, iii) tailoring the interfaces between electrodes and electrolyte, iv) optimizing the composition of electrolytes and v) improving separating membranes to enhance their stability and prevent the crossover of Br species. This section summarizes representative new findings of these five aspects.

### 4.1 Anodes

**Figure 9:**
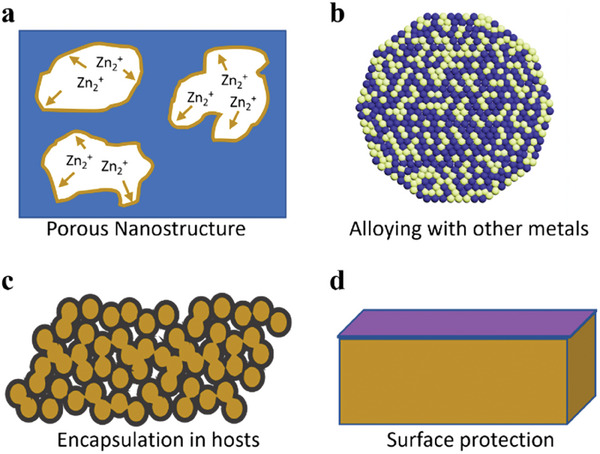
Schematic illustration of approaches used to minimize Zn dendrite formation and Zn dissolution in electrolytes: a) forming porous Zn structures, b) alloying with other metals, c) encapsulating Zn metal in a porous host, and d) creating a surface protection layer.

Zn plating must pass specific nucleation energy barriers on current collectors. As summarized in previous sections, the nucleation barrier leads to regions with higher energy batteries, resulting in preferential Zn deposition and dendrite growth. Several approaches have been explored to homogenize Zn platting, such as forming porous Zn structures, alloying Zn with other metals, encapsulating Zn metal in a host, creating a surface protection layer, as well as adding additives in electrolytes, which have led to enhanced cyclic life and CE. **Figure** **9** illustrates an overview of these approaches.

#### 4.1.1 Forming Porous Zn Structures

Commonly used metal foil anodes have a limited exposed surface area, making them prone to dendrite formation, especially under high current densities. Introducing porosity in metal anodes can increase electrode/electrolyte interfaces and lower specific area current densities, which may help stabilize the anodes. In comparison, nanoparticles can also provide a high specific surface area; however, nanoparticles tend to agglomerate during cycling. In particular, hierarchical porosity is desirable, providing well‐connected pore structures with large openings (pores). Such pore structures can provide not only large electrode/electrolyte interfaces but also enable structural stability upon deep discharge. Several methodologies have been reported to create Zn anodes with hierarchical porosity, including high‐temperature calcination, reduction‐induced decomposition, and solid‐state synthesis. For example, Wang et al. used the reduction‐induced decomposition method to reduce ZnCl_2_ into metallic Zn.^[^ ^99^ ^]^ The ZnCl_2_ crystals were exposed to naphthalenide solution, which selectively dissolved Cl^−^ from ZnCl_2_ crystal interfaces, resulting in a Zn monolith with a diameter of ≈200 µm. Although this method is a simple chemical process, scalable production of Zn monolith needs to be done in an Ar environment, limiting its applications.

**Figure 10:**
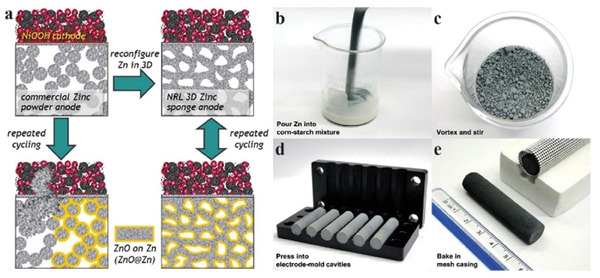
a) Schematic illustration of Zn deposition on a 3D Zn foam anode in a recharging Ni–Zn battery. Reproduced with permission.^[^ ^102^ ^]^ Copyright 2017, American Association for Advancement in Science. Photos showing the steps in the low‐cost synthesis of Zn sponge. b) Pour Zn powders into a mixture containing water, corn starch, and cellulose gum. c) Vortex while stirring the mixture. d) Press the Zn paste into electrode‐mold cavities. e) Place dried preforms into a mesh casing for baking: (center) baked 5 cm‐long Zn sponge; (right) mesh casing filled with Zn sponges. Reproduced with permission.^[^ ^104^ ^]^ Copyright 2020, Royal Society of Chemistry.

Several studies have used high‐temperature calcination methods to create porous Zn structures. Rajaei et al. presented a high‐temperature calcination method to achieve Zn foams with a high young modulus.^[^ ^100^ ^]^ Zn precursors were first molten at 450 °C in an Ar environment, followed by the addition of Ca and TiH_2_ for reduction and pore formation. Drillet et al. utilized a single‐step high‐temperature calcination method.^[^ ^101^ ^]^ The pore volume of Zn foam was increased by 20% when the alkaline content in the precursors was increased from 35 to 55%. Parker and coworkers thoroughly investigated a multi‐step calcination method to create 3D Zn foams for Zn batteries, efficiently suppressing the dendrite formation and achieving excellent reversibility (**Figure** **10**a).^[^ ^102^, ^103^, ^104^ ^]^ Zn particles without any additive were first calcinated at a temperature slightly lower than the melting point of Zn to fuse them together. The subsequent calcination in air at 600 °C provided the resulting ZnO@Zn structure necessary mechanical strength. The top surface of ZnO@Zn was then reduced back to Zn by electrochemical reduction. Subsequent studies showed that a third electrochemical reduction was not required, and ZnO@Zn can be directly utilized in Zn batteries where the thin ZnO surface layer was dissolved during the first few electrochemical cycles. This method provided a scalable approach to producing 3D Zn foams (Figure 10b). In a recent study, Parker et al. further investigated the formation of highly dense Zn foams by optimizing the composition of precursor materials.^[^ ^105^ ^]^

#### 4.1.2 Encapsulating Zn Metal in a Host

**Figure 11:**
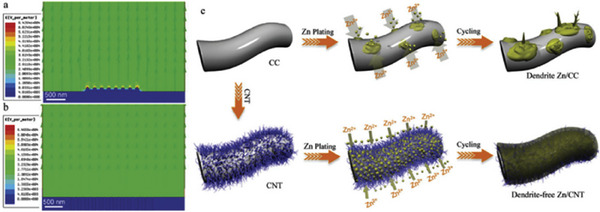
Simulated electric field distributions on a) a CC electrode and b) a CC@CNT electrode. The schematic illustrations of Zn deposition on CC and CC@CNT electrodes. Reproduced with permission.^[^ ^106^ ^]^ Copyright 2019, Wiley‐VCH.

Various porous templates have been used as hosts for Zn anodes to enable homogeneous Zn deposition during stripping/plating. Carbon materials, such as graphene, graphite, carbon nanotubes, and carbon cloth, are the most widely used host materials because of their high surface area, tunable pore sizes, excellent chemical and electrochemical stability, and ease of electronic property tuning. For example, Zheng et al. reported carbon cloth (CC) decorated with carbon nanotubes (CC@CNT) as a substrate for Zn plating/stripping, as shown in **Figure** **11**. The electric field distribution analysis revealed islands with a concentrated electric field that supported dendrite growth when CC was used as a substrate alone. In comparison, CC@CNT exhibited homogeneous electric field distribution and provided many exposed surfaces for stable Zn stripping/plating.

**Figure 12:**
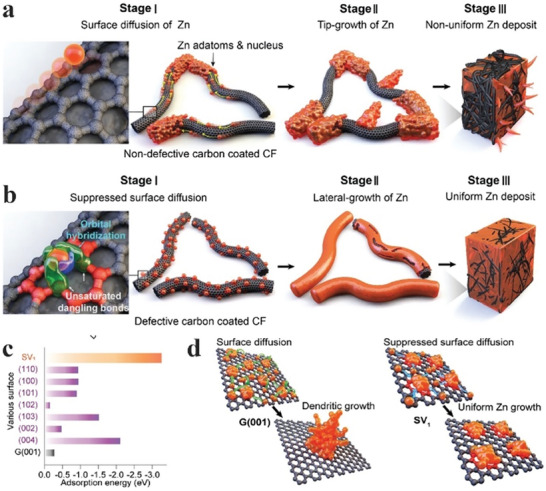
Schematic illustrations of a) Zn aggregation and subsequent non‐uniform Zn growth on non‐defective 3D carbon fibers and b) uniform Zn nucleation and lateral growth on defective carbon layer‐coated 3D carbon fibers. Comparison of adsorption energies for Zn adatoms on different Zn crystal planes, SV1, and the G (001) surface. j) Schematic representations of Zn nucleation and growth on the defect‐free graphene‐like surface and an SV1 defect‐containing carbon surface. Reproduced with permission.^[^ ^107^ ^]^ Copyright 2020, the Royal Society of Chemistry.

Pristine carbon surface is hydrophobic and has poor interactions with aqueous electrolytes. Non‐defective carbon surface promotes the diffusion of Zn ions toward its edge, followed by the nucleation of Zn ions preferentially on the tip of carbon sheets or fibers, as shown in **Figure** **12**a.^[^ ^107^ ^]^ Such depositions eventually lead to non‐uniform Zn depositions that promote dendrite formation. Lee et al. recently designed a novel defect‐rich carbon fiber‐based host for Zn where the defect‐rich host offered single vacancy defects.^[^ ^107^ ^]^ It was found that the sp^2^ orbitals in single vacancy defects overlapped with Zn orbitals which provided the basis for adsorption and subsequent nucleation of Zn ions at localized sites on the defective carbon (Figure 12b). Such nucleation prevented the diffusion of Zn ions toward the tip or edge of the carbon structure. Thus, the nuclei present on the carbon surface merged with neighboring nuclei to provide a homogeneous covering of Zn on the carbon surface.

Although the nucleation of Zn was found to be better on the defective carbon surfaces, it is unclear which type of defects would most favor homogeneous Zn nucleation. Lee et al. further calculated the adsorption energies of Zn on different defective surfaces.^[^ ^107^ ^]^ In addition to pristine graphite surface having no defects, they considered four different types of defects: single vacancy, stone‐wales, and two‐double vacancy. The Zn adsorption energy is much higher on single vacancy defects (≈ 3.26 eV) than on graphite surfaces (0.27 eV), stone wales (0.24 eV), and double vacancy defects (0.24 eV), as shown in Figure 12c. This result indicated that single vacancy defects would bind Zn ions stronger than other defects. Understanding how Zn deposition proceeds after the initial nucleation on defect‐free and defect‐rich carbon surfaces is also essential. The adsorption energy of Zn ions on Zn planes is much smaller than that on single vacancy defects. Without defects, Zn would prefer to plate on already nucleated islands over defect‐free carbon surfaces, promoting dendrite formation. In contrast, Zn would plate more homogeneously in the presence of defects, as summarized in Figure 12d. Carbon electrodes with single vacancy defects were synthesized using metal‐organic frameworks as a sacrificial template, demonstrating an excellent areal capacity of 15 mAh cm^−2^ at a high current density of 100 mA cm^−2^ in ZBBs. The electrodes effectively suppressed the dendrite growth, demonstrating stable performances over 5000 cycles with >97% CE.

### 4.2 Cathodes

**Figure 13:**
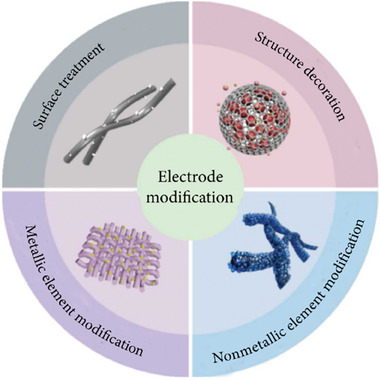
An overview on cathode modification methods for ZBBs. Reproduced with permission.^[^ ^30^ ^]^ Copyright 2022, American Association for the Advancement of Science.

Cathodes in ZBBs face several issues, including sluggish Br_2_ redox reactions, a highly corrosive environment, and high volatility of Br species. Various methods have been explored to modify cathodes to address these issues, including surface treatment, structural decoration, and the incorporation of catalytically active metallic and nonmetallic species, as illustrated in **Figure** **13**.^[^ ^30^ ^]^ Some incorporated materials suffer from various additional issues, limiting their adoption. For example, metal and metal oxides, such as Pt, ZrO_x_, TiO_x_, and AlO_x_ can offer active catalytic sites for Br_2_ redox reactions but suffer from poor stability. Porous carbon materials provide high electrical conductivity and a large surface area for the adsorption of Br species. However, they have relatively low catalytic activity for Br_2_ redox reactions and induce concentration polarization in electrolytes.^[^ ^29^, ^31^, ^41^ ^]^ In this section, details of these methods are described.

**Figure 14:**
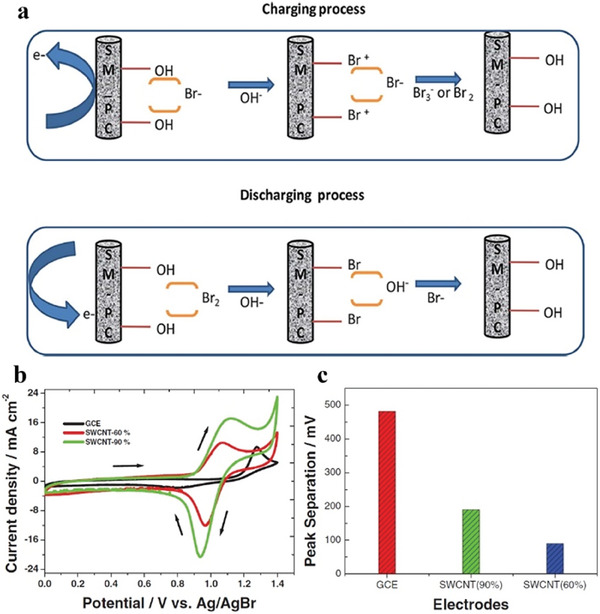
a) Schematic illustration of a proposed reaction mechanism of Br_2_/Br^−^ redox reaction on functionalized carbon paper. Reproduced with permission.^[^ ^108^ ^]^ Copyright 2018, Wiley‐VCH. b) Cyclic voltammetry of GCE, SWCNT (60%)/GCE, and SWCNT (90%)/GCE at a scan rate of 20 mV s^−1^ in 0.05 m ZnBr_2_ +1.0 m HClO_4_, and c) peak separation in (b) versus different electrodes. Reproduced with permission.^[^ ^25^ ^]^ Copyright 2013, IOP.

Carbon materials have good corrosion resistance in Br‐containing electrolytes. They offer good structural and chemical property tunability and can also act as a host to load other active nanomaterials. Suresh et al. used carbon paper as a cathode in ZBFBs and studied Br_2_ redox reactions on carbon surfaces.^[^ ^108^ ^]^ Carbon paper was treated with H_2_SO_4_ to introduce hydroxyl and carboxylic groups to improve its surface hydrophilicity. Hydrophilic carbon surface showed improved interactions with Br ions, resulting in enhanced electrochemical activity (**Figure** **14**a,b). Munaiah et al. studied single‐walled carbon nanotubes (SWCNTs) as cathode materials in ZBBs.^[^ ^25^ ^]^ High‐purity SWCNTs exhibited better electrochemical activity than glassy carbon electrodes (GCEs) due to their exposed basal and edge planes and larger surface area. A cathode with a higher SWCNT content (90%) delivered ≈48% higher electrochemical response than that with 60% SWCNT content (Figure 14b,c).

**Figure 15:**
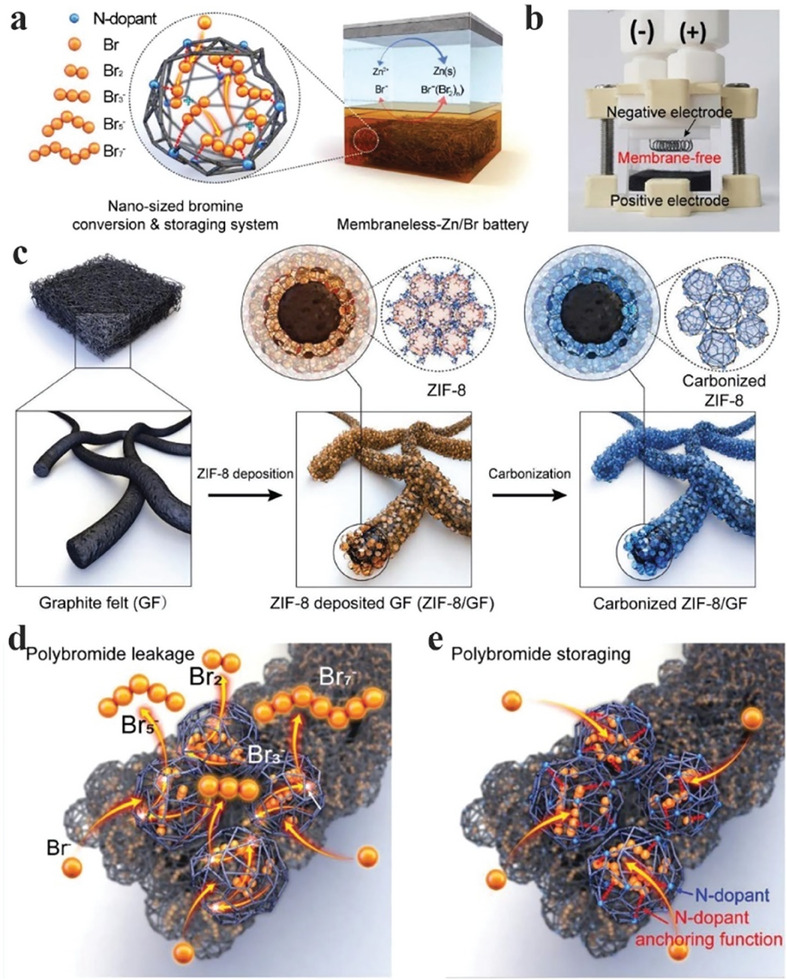
Carbon nanomaterials trapping Br species for MLFL‐ZBBs: a) Schematic illustration of an MLFL‐ZBB employing N‐rich carbon cages as cathode materials to trap Br species. b) A photo of the MLFL‐ZBB before charging. Structural and chemical characteristics of graphite felt (GF) and ZIF‐8/GF. c) Schematic illustration of the synthesis of carbonized ZIF‐8/GF. Schematic illustration of different Br species capture and storage mechanisms for d) ZIF‐8/GF carbonized at 1000 °C and e) ZIF‐8/GF carbonized at 700 °C. Reproduced with permission.^[^ ^58^ ^]^ Copyright 2019, Wiley‐VCH.

Doping heteroatoms in a carbon framework may create active catalytic sites for Br redox reactions.^[^ ^30^, ^109^ ^]^ For example, Xiang et al. used an ammonia‐assisted methodology to synthesize the N‐doped carbon as cathode materials in ZBBs.^[^ ^29^ ^]^ A high voltage (≈83%) and energy (≈82.5%) efficiencies were achieved at a high current density of 80 mA cm^−2^ with excellent stability for over 200 cycles. Similarly, Lee et al. utilized metal‐organic framework (MOF) derived N‐rich carbon cages to trap Br_2_ and polybromide ions, defined by the authors as a “nanosized bromine conversion and storage system”, as shown in **Figure** **15**a,b.^[^ ^58^ ^]^ Polybromine anions show much more negative adsorption energies on N‐doped carbon (−4.2 eV) than on undoped graphitic surfaces (−0.047 eV). In another study, N‐doped carbon derived from ZIF‐8 particles was grown on graphite felt 3D mesh, followed by subsequent carbonization, as shown in Figure 15c. The highest pyridinic N content was obtained under 700 °C carbonization, where pyridinic N constituted nearly ≈50% of total N. The authors further demonstrated that undoped carbon provided pathways that allow Br ions to escape, causing self‐discharge and poisoning of anodes. In contrast, N‐doped carbon materials obtained at different carbonization temperatures efficiently blocked Br ion diffusion (Figure 15d,e).

In ZBBs, polarization manifests as a voltage disparity between the expected and observed performance arising from internal resistance and ion transport constraints. This phenomenon leads to a reduction in both efficiency and capacity, accompanied by heat generation. Consequently, heightened polarization in ZBBs, mainly attributable to a Br_2_ cathode, can result in a decrease in energy density and an overall decline in efficiency. Notably, Lee et al. investigated the influence of the carbon host on polarization and voltage efficiency.^[^ ^58^ ^]^ In an FL‐ZBB without membrane, voltage efficiency attenuated as the discharge current density increased, primarily attributed to the escalation in polarization. Additionally, when assessing the charge and discharge polarizations of nitrogen‐doped carbon felt (NGF) synthesized at 1000 °C (denoted as NGF‐1000), NGF‐1000 was considerably smaller than those exhibited by NGF‐700 and GF. While NGF‐700 displayed slightly inferior voltage efficiencies compared to NGF‐1000, both NGF‐700 and NGF‐1000 demonstrated superior voltage efficiency when contrasted with GF. These findings indicated a beneficial impact of microporosity on cathode kinetics. The reduced polarizations and enhanced voltage efficiency associated with NGF‐1000 and NGF‐700 can be ascribed to their improved microporous structure, which is likely to facilitate more efficient ion transport and electrode reactions, consequently contributing to an overall enhancement in battery performance.

**Figure 16:**
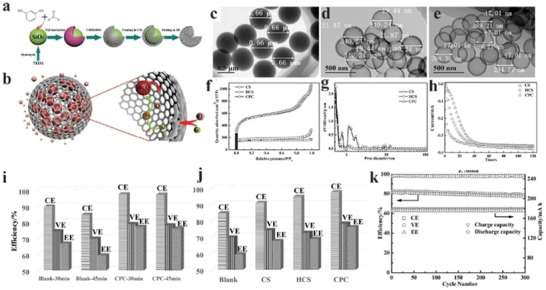
a) Schematic illustration of the synthesis of cage‐like porous carbon using SiO_2_ templates and b) trapping of Br species by cage‐like porous carbon (Q stands for the complexing agent and BrnQ stands for Br_2_ complexes). c–e) TEM images of c) carbon sphere (CS), d) hollow carbon sphere (HCS), and e) cage‐like porous carbon (CPC). f) N_2_ sorption isotherms curves of CS, HCS, and CPC. g) Pore size distribution curves of CS, HCS, and CPC. h) I_ring_–t curves of different carbon materials in 2 m ZnBr_2_ + 0.4 m MEPB electrolyte. i) The efficiency of Blank ZBFB and ZBFB with CPC at different charging times. j) The efficiencies (Coulombic efficiency (CE), voltage efficiency (VE), and energy density (EE)) of ZBFBs with different cathode materials at 80 mA cm^−2^. k) The efficiency and capacity of ZBFBs using CPC as cathode materials at 80 mA cm^−2^. Reproduced with permission.^[^ ^110^ ^]^ Copyright 2017, Wiley‐VCH.

Despite the improved adsorption capacity of N‐doped carbon materials, Br species may still escape from them. Researchers have explored carbon materials with cage‐like porous structures to prevent Br species’ leaking further. For example, Wang et al. used silica particles as a template and coated them with a polymer coating.^[^ ^110^ ^]^ After calcination, silica particles were etched using HF, resulting in cage‐like porous carbon (CPC) to host Br species, as shown in **Figure** **16**a,b. Figure 16c–e shows several carbon materials with a round shape with a diameter of ≈500 nm. CPC has a high specific surface area of 1665 m^2^ g^−1^ with a narrow pore size distribution ≈1.1 nm (Figure 16f–h). Its pore size is close to the molecular size of Br^−^ ions, MEP^+^, and bromine complex (MEPBR3) at 0.483, 0.925, and 1.24 nm, respectively. The bromine complex is slightly larger than CPC's pores, preventing their escape from CPC. Cathodes made of CPC exhibited excellent CE at various charging intervals. They stabilized ZBBs for over 300 cycles with a high CE (Figure 16i–k).

### 4.3 Electrode/Electrolyte Interfaces

Electrode/electrolyte interfaces play a crucial role in the reversibility of ZBBs as Zn ions travel through them reversibly. A solid electrolyte interphase (SEI) layer is often formed on electrode surfaces due to the decomposition of electrolytes during the charge/discharge process. The chemistry of electrodes and electrolytes, along with applied potentials, determines the composition and stability of electrode/electrolyte interfaces. An unstable interface would cause continuous degradation and formation of SEI layers that consume electrolytes, leading to electrolyte shortage and low CE. Various additives have been added to electrolytes to stabilize the interface. For example, Zhao et al. proposed a pyridinium‐based additive to stabilize Zn deposition because of the presence of electron‐donating groups that provided a lower positive redox potential than Zn/Zn(II).^[^ ^111^ ^]^ Similar results were also reported by Easton et al. using 1‐ethylpyridium bromide as an additive.^[^ ^112^ ^]^

**Figure 17:**
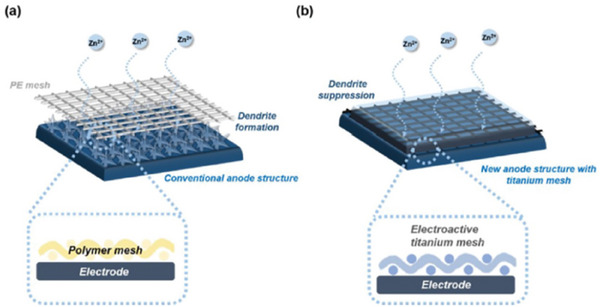
Schematic illustration of a) a typical anode with polymer mesh and b) a new anode with an electroactive Ti mesh. Reproduced with permission.^[^ ^114^ ^]^ Copyright 2021, Nature Publishing Group.

Modifying electrode surfaces can tune electrode/electrolyte interfaces. A common approach is to add a surface protective layer between electrodes and electrolytes to prevent undesirable side reactions and regulate ionic distribution in many other types of batteries. Various organic, inorganic and composite surface protection layers have been reported for this purpose.^[^ ^113^ ^]^ However, few studies have reported Zn surface protection layers for ZBBs. Recently, Lee et al. stabilized Zn anodes in ZBBs by replacing the traditional polymer mesh with a Ti mesh.^[^ ^114^ ^]^ It was proposed that the polymer mesh reduced the effective surface area of Zn anodes, limiting the charging current density (**Figure** **17**a). The Ti mesh provided abundant active sites, additional electroactive surface area, higher mechanical strength, and channels for fast ion transport (Figure 17b).

Electrode/electrolyte interface modification has also been carried out on cathodes. Xu et al. reported a membrane‐free ZBB in which Br_2_ dissolved in CCl_4_ was used as cathode materials.^[^ ^115^ ^]^ The immiscibility between the aqueous electrolyte of ZnBr_2_ and CCl_4_ avoids mixing electrolytes and cathodes. Br_2_ generated from redox reactions was contained by CCl_4_, leading to limited or no crossover. And no solid cathodes and separators were required. The battery delivered a CE of ≈96% and energy efficiency of ≈81% at a current density of 15 mA cm^−2^.

### 4.4 Electrolytes

Electrolytes stabilize electrode/electrolyte interfaces, determine the working voltage, and control ionic conductivity, which influences Zn deposition on anodes, redox reactions, and complexation of Br species on cathodes. A low‐concentration electrolyte would result in higher internal resistance.^[^ ^57^ ^]^ However, the commonly used ZnBr_2_ electrolyte has the highest conductivity when the molar concentration of ZnBr_2_ is at 2 m. On the other hand, Br_2_ can dissolve in an aqueous ZnBr_2_ solution at a much higher concentration (7 m). Increasing ZnBr_2_ concentration would improve the Br_2_ solubility. Br_2_ gas may be formed during the charging process, which is volatile, toxic, and corrosive. Higher ZnBr_2_ concentration would increase Br crossover, leading to anode poisoning and self‐discharge. The trade‐off between ZnBr_2_ molar concentration and Br_2_ dissolution needs to be balanced.^[^ ^116^ ^]^ In ZBFBs, quaternary complexing agents are usually added to the cathode side to fix Br ions, following the reaction:

(5) $$2{\mathrm{Br}}^{-}+{\mathrm{QBr}}^{-}\to {\mathrm{QBr}}^{3-}+2e^{-}$$

Various complexing agents have been explored, as summarized in **Table** **3**. Their roles in ZBFBs have also been summarized elsewhere.^[^ ^15^ ^]^

**Table 3:** Br_2_ complexing agents used in ZBFBs.

Complexing agents	Chemical structures	References
N‐Ethyl‐N‐methylpyrrolidinium bromide (1‐ethyl‐1‐methylpyrrolidin‐1‐ium bromide, MEP)	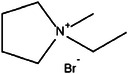	[116, 117, 118, 119]
N‐Chloro‐N‐methylpyrrolidinium bromide (1‐chloro‐1‐methylpyrrolidin‐1‐ium bromide, CMPB)	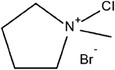	[120]
N‐Methoxymethyl‐N‐methylpipperidinium bromide (1‐(methoxymethyl)−1‐methylpiperidin‐1‐ium bromide)	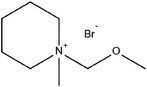	[121, 122]
Polydiallydimethylammonium bromide	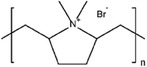	[123]
1‐(2‐Hydroxyethyl)−3‐methylimidazolium bromide (2‐(3‐methylimidazol‐3‐ium‐1‐yl)ethanol bromide, [C2OHMIm]Br)	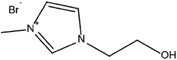	[124]
Dimethyldiethyl ammonium bromide (diethyl(dimethyl)azanium bromide, 2M2E)	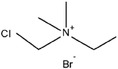	[120]
Tetrabutylammonium bromide (tetrabutylazanium bromide, TBB)	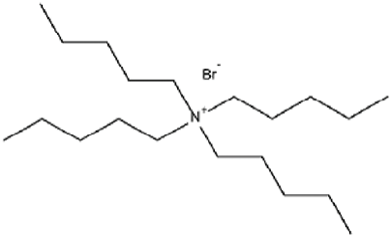	[125, 126, 127]
1‐(2‐hydroxyethyl)‐pyridinium bromide	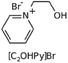	[124]

**Figure 18:**
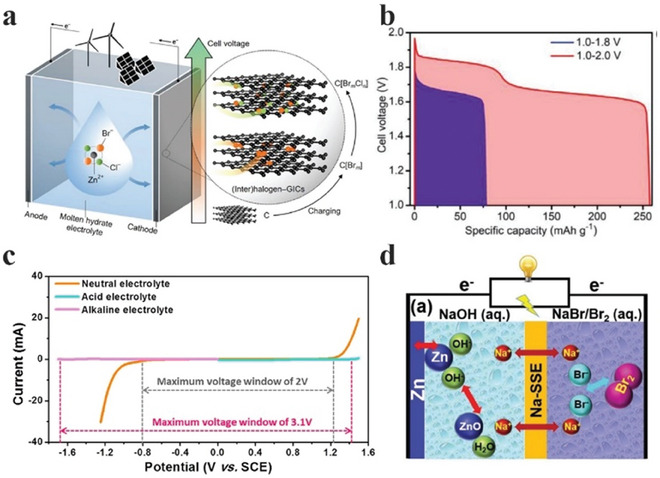
a) Schematic illustration of the cell structure and working principle of zinc–dual‐halogen battery using a molten hydrate electrolyte. b) Comparison of the discharge profiles involving single‐halogen or dual‐halogen redox couples. Reproduced with permission.^[^ ^134^ ^]^ Copyright 2020, Wiley‐VCH. c) The electrochemical stability windows of an alkaline electrolyte (2 m KOH) for the anode and an acid electrolyte (0.5 m H_2_SO_4_) for the cathode in comparison with a neutral electrolyte (1 m K_2_SO_4_) using a graphite rod as the working electrode, a Pt wire as the counter electrode, and a saturated calomel electrode as the reference electrodes. Reproduced with permission.^[^ ^135^ ^]^ Copyright 2019, Elsevier. d) Schematic illustration of a Zn–Br_2_ battery with a mediator‐ion solid‐state electrolyte in a Zn(NaOH)//Na‐SSE//Br_2_ (NaBr) cell. Na‐SSE refers to Na^+^‐ion solid‐state electrolyte. Reproduced with permission.^[^ ^136^ ^]^ Copyright 2017, Wiley‐VCH.

Additives have been used to tune the properties of electrolytes for ZBBs.^[^ ^128^ ^]^ For example, methanesulfonic acid was added to improve ionic conductivity and suppress Zn dendrite formation.^[^ ^129^ ^]^ Polyoxyethylene sorbitan monolaurate was added to enhance the mixing of the catholyte.^[^ ^130^ ^]^ Polyoxyethylene assisted Br_2_ reduction during the discharge. Recently, dual halogen electrolytes were reported to increase the working voltage window and provide higher energy density due to the combination of different redox reactions.^[^ ^131^, ^132^, ^133^ ^]^ For instance, Liu et al. reported a Br and Cl‐containing molten hydrate electrolyte.^[^ ^134^ ^]^ The dual ion electrolyte enabled the possibility of ion intercalation in carbon materials used at the cathode side (**Figure** **18**a). It also extended the voltage window from ≈1.8 to ≈2.0 V, resulting in two distinct plateaus, corresponding to the redox potentials of Cl and Br, which increased the energy storage capacity from ≈75 mAh g^−1^ based on common aqueous electrolytes to ≈250 mAh g^−1^ (Figure 18b). In another study, Yu et al. combined an alkaline (2 m KOH) electrolyte and an acidic (0.5 m H_2_SO_4_) electrolyte to extend the voltage window up to ≈ 3.1 V (Figure 18c).^[^ ^135^ ^]^

In addition to electrolyte compositions, the circulation of electrolytes also strongly impacts the performance of ZBFBs. Yang et al. studied the role of electrolyte circulation speed. They found that variable flow rates led to Zn dendrite formation on the anode side and the separation of polybromides from the aqueous phase electrolyte.^[^ ^137^ ^]^ They concluded that electrolyte circulation should be minimal to avoid the poor mixing of polybromides and the aqueous electrolyte in ZBFBs. An essential new electrolyte development is to develop solid‐state electrolytes to suppress dendrite formation and Br_2_ crossover. For example, Yu et al. reported a Na‐ion solid‐electrolyte that can couple different battery chemistries, such as Zn or Fe anodes and Br_2_ and potassium ferricyanide cathode (Figure 18d).^[^ ^136^ ^]^ The redox reactions are sustained by shuttling mediator Na ions in the solid electrolyte.

### 4.5 Separators

**Figure 19:**
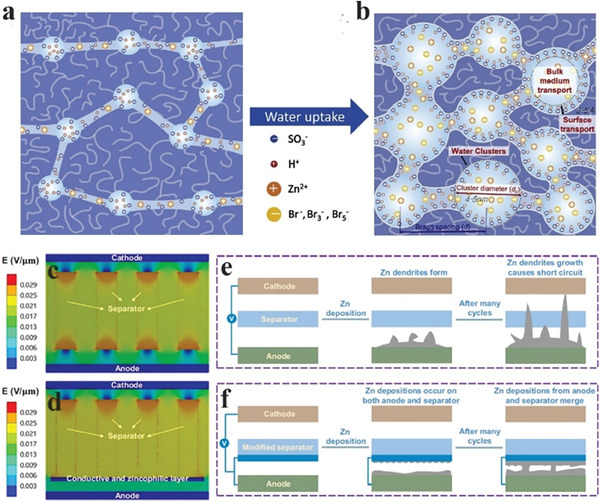
Water cluster structures at different hydration levels. a) The formation of small water clusters at a low hydration level; negatively charged ions cannot readily go through the narrow ion‐conducting channel. b) Large water clusters at a high hydration level allow both positive and negative charges to go through freely. Reproduced with permission.^[^ ^142^ ^]^ Copyright 2018, Elsevier. Theoretical calculation and protection mechanism of a modified separator. c) Electric field distribution with the pristine separator. d) Electric field distribution in the non‐contact region of the modified separator and the anode. e) Schematic illustration of Zn deposition with the pristine separator. f) Schematic illustration of Zn deposition in the contact region of the modified separator and the anode. Reproduced with permission.^[^ ^143^ ^]^ Copyright 2021, Nature Publishing Group.

An effective separator with good mechanical strength and suitable chemistry can suppress dendrite formation and prevent Br_2_ crossover.^[^ ^138^, ^139^ ^]^ The chemistry and porosity of separators have been optimized to improve the performance of ZBBs. SF600 or Daramic membranes (porous polyethylene membranes with hydrophilic treatments) have been widely used as separators in commercial ZBFBs. In comparison, ion‐exchange membranes (e.g., Nafion) are relatively denser and have shown better Br_2_ blocking capability.^[^ ^140^, ^141^ ^]^ For instance, Lai et al. found the CE of ZBBs using Nafion was ≈15% higher than those using Daramic. However, they found that the Nafion separator lowered the energy efficiency due to its higher resistance.^[^ ^119^ ^]^ Recently, Kim et al. showed that ion‐exchange membrane resistance decreased by tuning the water cluster size in membranes.^[^ ^142^ ^]^ The bi‐ionic transport was enabled in the Nafion membrane by scaling the water cluster size during the pretreatment under high temperatures. Narrow channels would restrict the anion transport due to nearby SO_3_^−^ ions; however, scaling these channels to a broader diameter would provide pathways for anion transport, as shown in **Figure** **19**a,b.

Adding another layer to separators may introduce desirable functional groups without changing their mechanical and transport properties. Zhang et al. coated a carbon layer on the separator facing the cathode side, which increased the electrochemical activity by decreasing the internal resistance.^[^ ^144^ ^]^ They achieved an energy efficiency of ≈75% at a 40 mA/cm^2^ current density. Hou et al. reported simultaneous modification of Zn metal surfaces and separators to suppress dendrite formation.^[^ ^143^ ^]^ The electric field distribution analysis suggested a nonuniform electric field distribution on the anode side spurred by pores in the separator (Figure 19c). The pores allowed fast diffusion of Zn ions, and the areas of the electrode directly adjacent to the pores are more likely to have Zn deposition and dendrite growth. Modifying the separator can homogenize the electric field distribution on the anode surface, stabilizing the subsequent Zn plating on the electrode (Figure 19d). The separator was modified with a thin layer of Sn using a sputtering technique and a doctor blade coating to achieve a relatively thicker coating. Using the Sn coating on the separator, stable performance was achieved for over 100 h at 5 mA cm^−2^ for 5 mAh cm^−2^ (Figure 19e,f).

## 5 Conclusion and Prospects

Rechargeable batteries, especially LIBs, have seen tremendous growth in the last few decades. However, recently, several alternative battery chemistries have shown the potential to complement or even replace some current battery technologies. ZBBs, although known for over 100 years, have regained attention again because they do not require scarce elements and offer safe operation with promising energy density. ZBBs have been primarily developed in flow battery configurations, requiring pumps to circulate electrolytes, which limits their potential applications. The recent development of FL‐ZBBs does not require additional pumps. All components can be sealed in a small container with different configurations, which opens new application opportunities from portable to large grid‐scale applications. Despite fast progress, ZBBs encounter many challenges, summarized in this review. For ZBFBs, apart from the requirement of pumps, the performance of Zn anodes is affected by dendrite formation, dissolution of Zn in electrolytes, and H_2_ gas formation from HER. Cathodes are affected by highly corrosive Br species and their crossover to anodes. Electrodes in FL‐ZBBs have similar issues. In addition, FL‐ZBBs risk electrolyte runout and gas accumulation in their containers. Many efforts have been devoted to addressing these challenges, including electrode composition and structure optimization, the use of Br complexing agents, and electrolyte additives. Although promising results have been obtained, there are a lot of areas for further improvement. We propose the following research areas to be the priority in the near term:

*Functional carbon‐based cathodes*. Carbon materials in cathodes play essential roles in enabling efficient redox reactions in ZBBs. They should process high conductivity, suitable porosity, excellent corrosion resistance, and abundant active sites for physical absorption and revisable redox reactions of Br species. These properties would, in turn, increase the charge transfer rate and ultimately improve the energy efficiency of ZBBs. The research efforts should focus on carbon structure designs and optimization of their surface functional groups. For example, porous carbon materials with cage‐like structures may provide confined spaces for confining the oily Br phase during the reversible charge/discharge. Functionalized carbon surface may catalyze the redox reaction of Br species.

*Robust Zn based anodes*. While extensive research has been conducted to stabilize Zn anodes, a significant need remains to extend their cyclic life. This is especially critical for developing FL‐ZBBs, where exceptionally stable anodes are required in strong acidic conditions. To achieve this objective, efforts should be directed toward mitigating dendrite formation on anodes, as this is paramount for preventing short circuits and enhancing battery safety. One approach involves exploring improved hosts for Zn's reversible stripping and plating, such as 3D porous hosts and various Zn metal morphologies. Enhancing compatibility between anodes and Br_2_‐containing cathodes is also crucial to obtaining good overall battery efficiency.

*Suitable electrolyte additives*. Functional additives can improve the stability and widen electrolytes' operational potential range. To date, many additives have been reported to be effective in different types of aqueous Zn‐ion batteries at mild acidic or neutral conditions. Some of these useful strategies may potentially be transplanted into ZBBs to regulate Zn plating and stripping behaviors, suppress dendrite growth, reduce side reactions, and improve electrolyte stability. Meanwhile, this optimization process should also consider electrolytes' pH and ionic conductivity.

*Solid and quasi‐soli (gel) electrolytes*. Using solid or quasi‐solid (gel) electrolytes to replace liquid electrolytes may suppress Br_2_ crossover inside ZBBs, reducing self‐discharge and protecting anodes. Moreover, they could also avoid electrolyte decomposition and enable safer operation due to their negligible volatility. Thus, developing novel solid or quasi‐solid (gel) electrolytes with porous and hierarchical structures with high ionic conductivity is a promising research direction for ZBBs.

*Robust membrane with high ion selectivity*: Membranes play an essential role in ZBBs to prevent Br_2_ crossover. Unstable membranes in current ZBBs compromise their cycle life. As a result, developing durable membranes with high ionic selectivity and conductivity in acidic electrolytes is a critical research topic for ZBBs.

ZBBs have gained increasing attention because of their attractive features, as summarized above. Various types of ZBBs have been commercialized. However, their broad adoption and commercial success still need to be determined. Significant research efforts are required to further improve their electrochemical performance and service life, as well as lower their costs. There are many exciting fundamental science questions and technical challenges waiting to be addressed.

## Conflict of Interest

The authors declare no conflict of interest.
